# Syringomyelia Associated with Chiari 1 Malformation in Adults: Positive Outcome Predictors after Posterior Fossa Decompression with Duraplasty

**DOI:** 10.3390/jcm12083019

**Published:** 2023-04-21

**Authors:** Palma Ciaramitaro, Giuseppe Migliaretti, Marilena Ferraris, Andrea Garnero, Giovanni Morana, Paolo Carucci, Ilaria Stura, Fulvio Massaro, Diego Garbossa

**Affiliations:** 1 Neuroscience Department, University of Torino, 10126 Torino, Italy; giovanni.morana@unito.it (G.M.); paolop.carucci@gmail.com (P.C.); ilariastura@gmail.com (I.S.); fulvio.massaro@fastwebnet.it (F.M.); diego.garbossa@unito.it (D.G.); 2 CRESSC, AOU Città della Salute e della Scienza di Torino, 10126 Torino, Italy; 3 Department of Public Health and Paediatric Sciences, University of Torino, 10126 Torino, Italy; giuseppe.migliaretti@unito.it; 4 Diagnostic Imaging Department, AOU Citta’ della Salute e della Scienza di Torino, 10126 Torino, Italy; marilenaferraris@hotmail.it (M.F.); andrea.garnero@edu.unito.it (A.G.); 5 Neurosurgery Unit, AOU Città della Salute e della Scienza di Torino, 10126 Torino, Italy

**Keywords:** syringomyelia, Chiari 1 malformation, PFDD, surgery, PC-MRI, outcome, adults

## Abstract

Background: Syringomyelia (Syr) in patients with Chiari 1 malformation (CM1) may be attributable to abnormal dynamics of cerebrospinal fluid (CSF) in the upper cervical segment; fourth ventricle enlargement has been reported in association with a worse clinical and radiological presentation, independently of the posterior fossa volume. In this study, we analyzed presurgery hydrodynamic markers to evaluate if their changes could be associated with clinical and radiological improvement after posterior fossa decompression and duraplasty (PFDD). As a primary endpoint, we aimed to correlate improvement in the fourth ventricle area with positive clinical outcomes. Methods: In total, in this study, we enrolled 36 consecutive adults with Syr and CM1 who were followed by a multidisciplinary team. All the patients were prospectively evaluated with clinical scales and neuroimaging, including CSF flow, the fourth ventricle area, and the Vaquero Index by using a phase-contrast MRI before (T0) and after surgical treatment (T1-Tlast, with a range of 12–108 months). The CSF flow at the craniocervical junction (CCJ), the fourth ventricle area, and the Vaquero Index changes were statistically analyzed and compared to the clinical and quality of life improvement after surgery. The good outcome prediction ability of presurgical radiological variables was tested. Results: Surgery was associated with positive clinical and radiological outcomes in more than 90% of cases. The fourth ventricle area significantly reduced after surgery (T0-Tlast, *p* = 0.0093), but no significant associations with clinical improvement were found. The presurgical presence of CSF flow at the CCJ was able to predict a good outcome (AUC = 0.68, 95% CI 0.50–0.87 and LH+ = 2.1, IC 95% 1.16–3.07) and was also significantly associated with post-surgical pain relief (rho = 0.61 and *p* = 0.0144). Conclusions: Presurgery CSF flow at the CCJ is proposed as a radiological marker with the ability to predict a positive outcome after PFDD in adults with syringomyelia and CM1. Measurements of the fourth ventricle area could be useful additional information for evaluating surgical long-term follow-up; further experience on larger cohorts is required to better define the prognostic yield of this radiological parameter.

## 1. Introduction

Syringomyelia (Syr) is a rare neurological disorder characterized by abnormal single or multiple fluid-filled cavities within the spinal cord that cause typical neurological signs and symptoms; this entity is most frequently associated with Chiari malformation type 1 (CM1), with variable frequency levels reported in the literature (up to 90%) [1]. As observed in an epidemiological study, Syr is mainly symptomatic (62%), and includes sensitive (70%) and motor deficits (60%), neuropathic pain (32%), and autonomic disorders (i.e., bladder dysfunction, 19%). Although pediatric forms are known, symptomatic Syr generally appears in adults (average age of 38 years), particularly in females (64%), and those with chronic and progressive disabilities [2]. The quality of life in Syr patients is generally lower than in the general population, being comparable with that of patients with heart failure or malignant tumors [3].

Despite advances and the availability of diagnostic methods and surgical treatments, Syr remains to be a challenging neurological disorder, particularly with regard to its pathogenesis and optimal management. For patients with Syr associated with CM1, surgical foramen magnum decompression is the treatment of choice [4,5,6]. It involves posterior fossa decompression (PFD), which can be performed along with C1 laminectomy, reconstructive duraplasty (PFDD), and tonsil shrinkage (PFDDC). Clinical improvement is the priority in surgical decision making in order to achieve good outcomes ranging from 61.5 to 93% [4]; it seems to be related to a better cerebrospinal fluid (CSF) hydrodynamic model at the cranio-cervical junction (CCJ) after surgical decompression. Nonetheless, there are few indications with high levels of evidence on surgical treatment and some patients who undergo surgery do not obtain clinical and symptomatic improvement.

An MRI is considered to be the diagnostic gold standard for CM1 and Syr that provides useful information for neurosurgical treatment planning. In CM1, it has been previously found that CSF dynamics within the fourth ventricle, which is surrounded by critical brainstem structures, is partially obstructed due to cerebellar tonsils and arachnoid adhesions. Seaman et al. [7] recently demonstrated that the fourth ventricle could be enlarged in CM1 independently of the lateral ventricle size and was associated with greater tonsillar descent. Fourth ventricle enlargement was associated with a worse clinical and radiological presentation, independently of posterior fossa volume, and the authors considered this morphological parameter to be a useful adjunct in assessing patients with CM1. Sakas et al. [8] highlighted that, after CCJ decompression, an increase of more than 20% in the sum of anterior and posterior cervical CSF flow velocities consistently preceded or coincided with a marked headache improvement. Liu et al. [9] demonstrated that Syr in patients with CM1 may be due to decreased circulation time and abnormal dynamics of CSF in the upper cervical segment. Wang et al. [10] showed that innate bony dysontogenesis in patients with CM1 contributed to tonsillar ectopia and exacerbated CSF flow obstruction. Moreover, the presence of a pressure gradient between Syr and subarachnoid spaces supports the perivascular space theory which has been used to explain Syr formation [11]; this theory has been confirmed by further studies on CSF dynamics with phase-contrast MRI (PC-MRI) and ultrasonography [12,13] as well in more recent experimental models [14,15,16].

On the basis of these considerations, the overall objective of this study was to confirm if presurgery hydrodynamic marker changes could be associated with clinical and radiological improvements after PFDD in adults with Syr and CM1; as a primary endpoint, we aimed to correlate the improvement in the fourth ventricle area with positive clinical outcomes.

## 2. Materials and Methods

### 2.1. Patient Population

Our study cohort was selected at the Interregional Centre of Expertise for Syringomyelia and Chiari Syndrome (CRESSC), Department of Neuroscience, Turin, using the CRESSC Database, a prospective database of information collected on adults and children with Syr and CM since 2010 (1062 patients, 749 female, 325 male, mean age 50.8 ± 16.8). This study was approved by the Local Ethics Committee (protocol no. 7837, 1 February 2010 and protocol no. 52554, 20 May 2015, Città della Salute e della Scienza di Torino Hospital, Turin). Informed consent was obtained from all individual participants included in this study. All patients underwent a multidisciplinary evaluation by the CRESSC team (neurologists, neuroradiologists, neurophysiologists, neurosurgeons, and physiatrists) in order to confirm a radiological and clinical diagnosis and to guide subsequent management (surgical or conservative). They were prospectively evaluated with clinical scales and neuroimaging (brain and spine PC-MRI including CSF flow), before and after surgical treatment.

In total, in this study, we enrolled 36 consecutive adults (25 females, 11 males, age range 18–70 years, average age 44 ± 15 years) with the Syr-CM1 complex (*n* = 26) or isolated CM1 (*n* = 10), diagnosed by the CRESSC Unit and surgically treated in the Neurosurgey Unit, Department of Neuroscience, Turin, between January 2010 and December 2020.

Patients were included if: (1) their age was ≥18 years at surgery; (2) they were diagnosed with the symptomatic CM1 or Syr-CM1 complex with clinical/neuroradiological evolutive trends and surgical indications [6]; (3) they were surgically treated by posterior fossa decompression and duraplasty [6,17]; (4) they underwent brain and spine PC-MRI, before and after surgery.

Subjects with asymptomatic and isolated CM1, or with prior posterior fossa decompression or hydrocephalus surgery were excluded. CM1 was diagnosed according to ICHD-3 radiological criteria for CM1 (IHS Classification, 2018) [18].

### 2.2. Surgical Techniques

Posterior fossa decompression with duraplasty (PFDD) was performed with the aim to restore normal CSF flow at the CCJ. In all patients, a small suboccipital craniectomy (3 × 3 cm), wide foramen magnum decompression, C1 laminectomy, and duraplasty (autologous dural patches) were performed. In the case of complete CSF flow obstruction at the CCJ or very low-lying tonsil herniation, a PFDD with tonsils coagulation (PFDDC) was preferred [6].

Multimodal intraoperative neurophysiological monitoring, according to standardized methodology [19], was carried out in all surgical interventions.

### 2.3. Follow-Up

MRI scans and clinical evaluations were performed presurgery (T0), post-surgery at 6 months (T1) and 12 months (T2), and then once a year for approximately 5 years following surgery (Tlast, range between 12 and 108 months, mean 55.25, SD 27.24).

In addition to neuroimaging studies, all 36 patients underwent neurological examination; disability, pain, and quality of life scales were administered at one time by a single neurologist (P.C.) at T0, T1, and T2. In the long-term outcome evaluation (T last), the results corresponded to 32 patients (4 patients lost at the follow-up).

### 2.4. Imaging Protocol and Analysis

MRI studies were performed on 1.5 T scanners (Signa GE, General Electric Medical Systems or Magnetom Aera, Siemens, Erlangen, Germany).

Tonsillar ectopia was quantitatively evaluated on brain sagittal Turbo Spin Echo (TSE) T1-weighted images (TR 421–560 ms, TE 8.4–12 ms, with a slice thickness of 3 mm). Measurements were taken on our Picture Archiving and Communicating System (Synapse, Synapse, Fujifilm Medical System, Morrisville, NC, USA).

The fourth ventricle area (FVA) was assessed on the same T1-weighted images. It was manually outlined on the midsagittal plane by a single trained reader (A.G.) to avoid interobserver variability, using the Synapse 3D Fujifilm’s software. MRI fourth ventricle size data were compared to a normal control group of 36 sex- and age-matched subjects that were admitted to our Neuroradiological Department.

The CSF flow study at the CCJ was conducted using the cine phase-contrast technique (TR 44.6–50 ms, TE 7–7.9 ms, with a slice thickness of 5 mm). A qualitative analysis of CSF flow at the posterior CCJ was performed with a dedicated CSF flow score, ranging from 0 (regular flow) to 3 (absent, Table 1a).

Syringomyelia size was quantified using the Vaquero Index (VI), defined as the ratio of the greatest width of the syrinx to that of the spinal canal at the same level [20], measured on spine sagittal TSE T1-weighted images (TR 450–460 ms, TE 9.8–12 ms, with a slice thickness of 3 mm). The presence of flow within the syrinx was assessed with the CSF flow study at the CCJ for syringobulbia or cervical syringomyelia and with spine sagittal spine T2-weighted images (TR 3010–3440 ms, TE 102–107 ms, with a slice thickness of 3 mm) for dorsal syringomyelia.

All the morphological variables (FVA, CSF flow at the CCJ, and VI) were measured before surgery (T0) and at each post-surgical timing (T1,T2, and Tlast).

### 2.5. Clinical Outcomes

Clinical signs and symptoms along with pain and disability scales were analyzed before and after surgery.

Clinical outcomes were evaluated using a dedicated score (clinical sign score (CSS)), including motor, sensory, and sphincter abnormal signs, and ranging from 0 (absence) to 3 (presence of all abnormal signs), as shown in Table 1b.

Headache and pain were quantified using VAS [21]; neuropathic pain was assessed by using a DN4 questionnaire and identified with a score ≥ 4 [22,23]. Disability was evaluated using the modified Rankin scale (mRS) [24]; the Beck Depression Inventory (BDI) [25] and the 36-Item Short Form Health Survey [26] were used to investigate the quality of life (QoL).

We defined improvement or stability based on at least 2 out of 3 clinical criteria (neurological signs, pain, and disability) and improvement in the QoL as positive outcomes.

### 2.6. Statistical Analysis

#### 2.6.1. Sample Size and Control Group Selection (the Fourth Ventricle Area)

Considering improvement in the fourth ventricle area from T0 (baseline) to Tlast to be the primary endpoint, on the basis of a “positive outcome” being defined as the improvement/stability of at least 2 out of the 3 clinical criteria, the sample power calculation was made; a sample of 36 patients (“cases”) and 36 healthy subjects (“normal controls”) gave a power rating of 83%, which assured good reliability [27].

In order to obtain fourth ventricle area normative data, 36 subjects with normal brain MRIs performed in our Neuroradiology Department were identified and matched to patients by sex and age. In the control group selection, a random match pair analysis was performed [28].

#### 2.6.2. Statistics

Means and standard deviations were used to describe continuous, presurgical demographic and clinical variables. Categorical variables were summarized by percentages and frequencies.

To evaluate changes in clinical and radiological variables, pre and post-surgery (T0-T1, T1-Tlast, and T0-Tlast), Friedmann and McNemar tests were used for continuous and categorical variables, respectively.

The prediction ability of the good outcomes of presurgical variables was tested based on the ROC curve estimation; the results were reported in terms of area under the ROC curve (AUC) and relative 95% CI [29]; for each variable, cut-off values with higher association with a positive outcome were assessed using the Youden method [30]. To validate the cut-off values as good outcome predictors, positive likelihood estimation (LH+) with a relative 95% CI was calculated [31]. Possible correlations between pre-surgery and post-surgery morphological measures were analyzed by estimating the non-parametric rho-Spearman correlation coefficient. The significance was tested at *p* < 0.05.

To evaluate the changes in morphological variables between groups (improved/non-improved), the logistic regression model was estimated; the results, in terms of odd ratios (OR), crude and adjusted for the principal confounding variables, were reported with a relative 95% CI. All the analyses were performed using the SAS^®^ Statistics Software.

## 3. Results

The demographic and phenotypic characteristics of all patients are reported in Table 2. Clinical and radiological variable changes, pre-surgery and post-surgery, in terms of frequency and percentages, are shown in Table 3.

### 3.1. Fourth Ventricle Area in Patients with CM1 ± Syr versus Normal Controls

**Figure 1:**
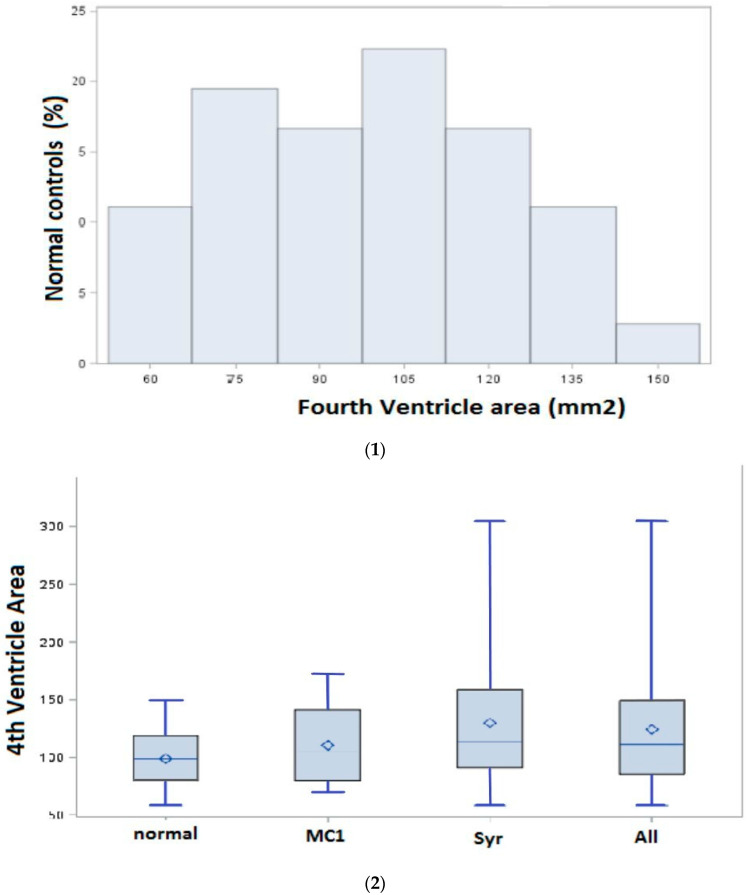
(**1**). **Fourth ventricle area in normal control group** (the graph shows the distribution of the fourth ventricle area (in mm^2^) in 36 normal controls, as measured in the midsagittal plane at T1-weighted MRI); (**2**) **fourth ventricle area in patients (presurgery) as compared with normal controls.** Boxplots of the fourth ventricle area show the shift in patients with Chiari 1 malformation (CM1), syringomyelia with CM1 (Syr), and all patients (All) at T0 (presurgery) compared with normal controls. There are significant differences in the mean fourth ventricle area between groups. The mean fourth ventricle area in Syr (129.8 mm^2^, SD 57.9, range 58–305, ***p* = 0.03**) Bold face characters indicate statistically significant differences and in All (124.2 mm^2^, SD 52.6, range 58–305, ***p* = 0.04**) is significantly increased compared with normal controls (98.7 mm^2^, SD 24.7, range 58–149). There was no significant difference in the mean fourth ventricle area in CM1 (110 mm^2^, SD 34.7, range 69–172) compared with normal controls (*p* = 0.38). Bold face characters indicate statistically significant differences.

The distribution of the fourth ventricle area in 36 controls (26 females, 10 males, mean age 41.5 ± 17.5) is shown in Figure 1(1). The mean fourth ventricle area was 98.7 mm^2^ (SD 24.7, range 58–149) in normal controls; in the patient subgroups, in the Syr subgroup, the mean fourth ventricle area was 129.8 mm^2^ (SD 57.9; range 58–305), and in the CM1, the mean fourth ventricle area was 110 mm^2^ (SD 34.7, range 69–172), as shown in Figure 1(2). In the presurgical (T0) MRI study, the fourth ventricle area was significantly increased in patients compared to normal controls (*p* = 0.04). The analysis, in all patients and stratified for diagnosis, showed a significantly increased fourth ventricle area in the “Syr” subgroup (Syr with CM1) versus normal controls before surgery (*p* = 0.0328).

After surgery (T1), the fourth ventricle area reduced, and, at long-term follow-up (Tlast), there was no significant difference between patients and normal controls (Table 4).

### 3.2. Follow-Up: Clinical and Radiological Outcomes

**Figure 2:**
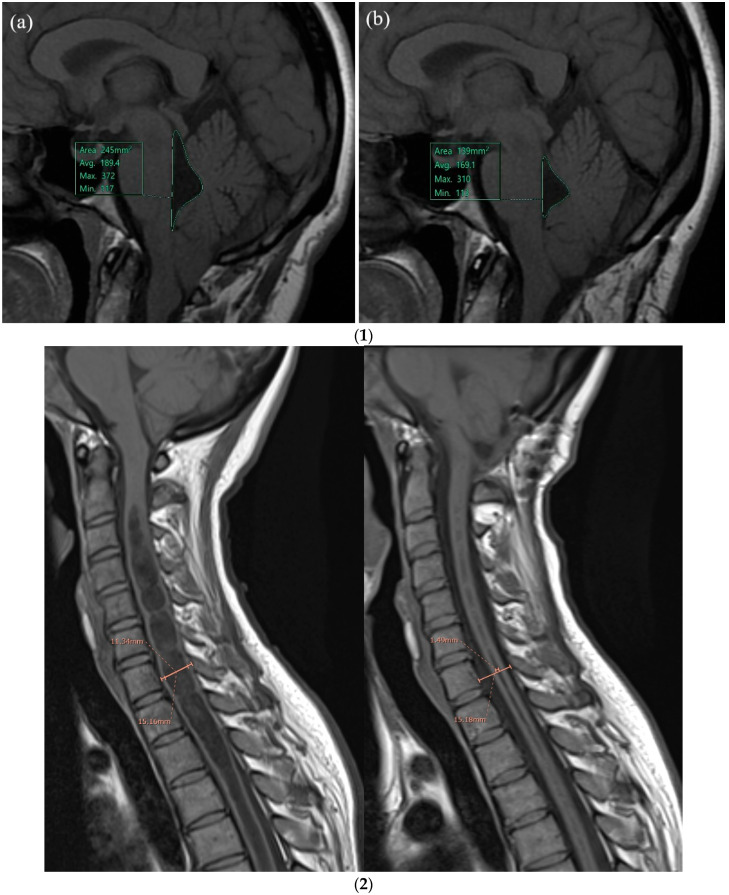
(**1**) **Illustrative case: Fourth ventricle area measurements, pre (a) and post (b) surgery:** 32-year-old female, Syr with CM1, pre (**a**) and post-surgery (**b**). Fourth ventricle area at T1-weighted MR imaging, acquired in the sagittal orientation. (**a**) Fourth ventricle area at T0 (presurgery) was 245 mm^2^ and (**b**) fourth ventricle area at T1 (post-surgery) was significant reduced (139 mm^2^). (**2**) **Illustrative cases: Vaquero Index measures pre- and post-surgery:** 32-year-old female, Syr associated with CM1. Pre (**a**) and post-surgery (**b**). The size of syrinx using the Vaquero Index (VI = ratio of the greatest width of the syrinx to that of the spinal canal at the same level, C7) in a midsagittal T1-weighted spine MR was measured. Syrinx size presurgery (VI = 0.75) was wide and strongly reduced after surgery (VI = 0.1).

The statistical pre/post-surgery comparisons among clinical and radiological variables are reported in Table 5. The VAS and CSS scores were significantly reduced, both at short-term and long-term follow-up (FU). The fourth ventricle area (Figure 2(1)) presented a significant reduction at short-term FU. The Vaquero Index (Figure 2(2)) was significantly reduced at short- and long-term FU; CSF flow at the CCJ was significantly improved at short- and long-term FU.

### 3.3. Quality of Life (QoL)

Before surgery (T0), we measured low scores in every SF-36 domain, significantly reduced compared with Italian normative values [32], as reported in Table 6.

After surgery, we found an improvement of QoL scores in all patients. In particular, the analysis showed a statistically significant difference in terms of SF-36 scores at short-term (physical SF-36, *p* < 0.05; mental SF-36, *p* < 0.005; total SF-36, *p* < 0.05) and long-term follow-up (mental SF-36, *p* < 0.05 and total SF-36, *p* < 0.05).

BDI had normal values before surgery, without worsening after surgery.

### 3.4. Positive Outcome Predictors

The CSF flow was shown to be a positive outcome predictor because of the AUC (AUC = 0.68, 95% CI 0.50–0.87) and the likelihood ratio (LH+ = 2.1, IC 95% 1.16–3.07). Patients with CSF flow at the CCJ before surgery (0–2 points at CSF flow score, Table 1) tend to have a higher probability (OR = 4.75, IC 95% 0.95–23.85, at the limit of significance) of post-surgical improvement than patients with absent CSF flow at the CCJ (3 points at the CSF flow score, Table 1). Moreover, presurgical presence of CSF flow at the CCJ was also significantly associated with post-surgical pain relief (r = 0.61 and *p* = 0.0144), particularly, in patients with neuropathic pain

**Figure 3:**
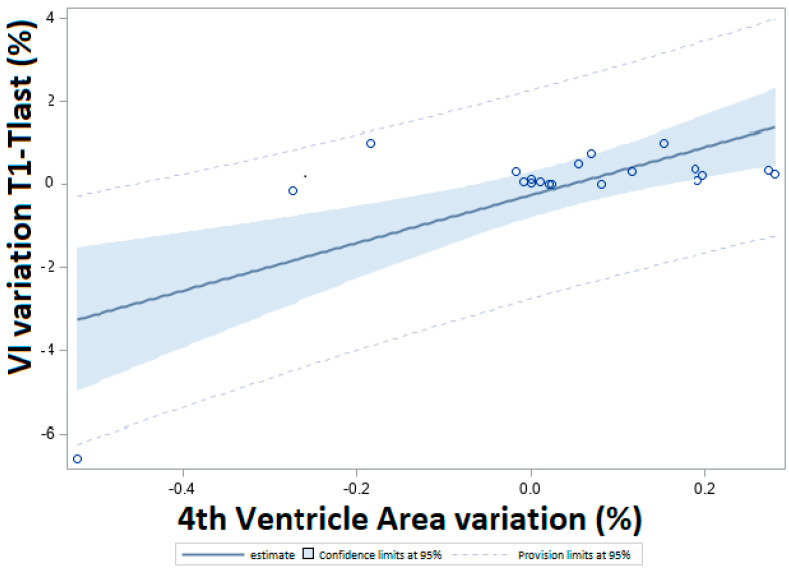
**Relationship between the variations of the Vaquero Index (VI) and the fourth ventricle area from T1 and Tlast.** Result of the linear regression between the variations of VI at T1-Tlast (in percentage, y-axis) and the fourth ventricle area (in percentage, x-axis). Circles are the single data, the line is the estimate, the blue area is the 95% CI, and the dotted lines are the provision limits. Slope of the curve = 1.16.

The fourth ventricle area was significantly correlated to the Vaquero Index in terms of changes (in percentages), mostly at long-term follow-up (T1-Tlast, r = 0.70, *p* = 0.0007). individual data points and a regression curve (slope = 1.16) are reported in Figure 3. There appears to be a positive association between the Vaquero Index and the fourth ventricle area changes and clinical scores, even if they do not reach statistical significance (OR = 2.19, 95% CI 0.18–26.09 and OR = 1.23, 95% CI 0.19–7.67, respectively).

All patients presented fourth ventricle area reduction and CCJ CSF flow improvement at long-term follow-up; moreover, in 78% of the patients, fourth ventricle area reduction was significantly associated with a CSF flow improvement at T1-Tlast (*p* < 0.05).

Among the demographic variables, age at surgery was able to predict a good outcome. In particular, undergoing surgery before 47 years of age was found to be a significant predictor of a good outcome (AUC = 0.71, 95% CI 0.54–0.89), increasing the probability of a good outcome by about six times (OR = 5.95, IC 95% 1.03–34.4).

## 4. Discussion

Surgery remains to be the most definitive treatment in symptomatic CM1 and Syr patients; the goal of surgery is to decompress the cerebellar tonsils to reduce the effects of tonsillar herniation through the foramen magnum and to restore normal CSF flow at the CCJ. In the last decade, important efforts have been made to share definitions, as well as diagnostic and therapeutic indications at the International level, both in adults and children [2,5,6,33,34]. In adults, posterior fossa decompression with duraplasty has shown a good clinical outcome in Syr associated with CM1 [35], also in long-term clinical and radiological follow-up. Although clinical improvement after PFDD seems to correlate with improved recanalization of CSF dynamics at the CCJ, the literature data on quantitative analysis of CSF dynamics a the CCJ in CM1 are few and controversial. Recently, Capel et al. [36] retrospectively observed an exclusive alteration in CSF dynamics at the foramen magnum plane accompanied by tonsils pulsatility; the authors also observed that surgery had a quantitative hemodynamic and hydrodynamic impact and proposed phase-contrast MRI as a useful additional tool for postoperative evaluation and follow-up.

In this pilot study, we prospectively evaluated the surgical outcome of a single-center adult population affected by Syr and CM1 undergoing PFDD, through short- (T1, mean 6 months) and long-term (Tlast, mean 55 months) follow-up, according to a multidisciplinary protocol, including PC-MRI study. The efficacy of surgery (good outcome) was quantified by standardized clinical and QoL scales (Rankin, VAS, SF-36, and BDI) and a dedicated clinical score (CSS, Table 1). Surgery was associated with a positive clinical outcome in more than 90% of cases, both in short- and long-term follow-up; we observed a significant clinical improvement at short-term follow-up after surgery, both in the Rankin scale and in the CSS score, which tended to persist over time; perceived pain (VAS) was also reduced immediately after surgery in more than 90% of patients, as shown in Table 3. QoL (SF-36 domains) significantly improved after surgery, both at short- and long-term follow-up, in all clinical phenotypes. From a radiological point of view, syrinx width reduced after surgery in more than 80% of patients at short-term follow-up and in more than 90% of patients at long-term follow-up. A young age at surgery (<47 years) was a significant good outcome predictor; according to these results, as also proposed by Aghakani et al. [37], surgery should be proposed as soon as possible in adults affected by syringomyelia with CM1.

For the first time, our study introduced the fourth ventricle area measurement on T1-weighted midsagittal MR images as a presurgical marker, describing this new technique and comparing patient samples with a normal control group (Figure 1(2)). In presurgical measurement, the fourth ventricle area was significantly enlarged compared to those of normal controls, particularly in Syr patients. After surgery, a statistically significant reduction in the fourth ventricle area of patients was observed. We did not measure the fourth ventricle volume. Changes in fourth ventricle volume in CM1 caused by partial obstruction of CSF flow due to tonsillar engagement in the foramen magnum have been reported by Seaman et al. [7]. The authors demonstrated a positive correlation between fourth ventricle volume and tonsillar descent and between fourth ventricle volume and clinical severity. Similarly, we also observed presurgical fourth ventricle area enlargement, more evident in patients with syringomyelia, with more severe clinical impairment than in isolated Chiari patients; post-surgical morphological variation of the fourth ventricle area was significantly associated with CSF flow improvement in our study, but not with a good clinical outcome.

A further issue in our patients concerns post-surgery positive correlations between a reduction in the fourth ventricle area and improvement of CSF flow at the CCJ and between fourth ventricle area and the Vaquero Index, indirect index of syrinx diameter and intramedullary tension [20]. In particular, a reduction in the fourth ventricle area after surgery was associated with a Vaquero Index reduction, at short-term follow-up and, even more, at long-term follow-up. Although clinical symptoms do not always correlate with the presence or extension of syrinx before surgery, permanent syrinx reduction at long-term follow-up after surgery may indicate adequate posterior fossa decompression and improved CSF dynamics at the CCJ and into the spinal subarachnoid spaces. Moreover, the evidence of a correlation between fourth ventricle area and Vaquero Index reduction further support the perivascular space theory, used to explain Syr formation [11].

In this study, the presence of CSF flow at the CCJ before surgery predicted (86% specificity) a positive outcome of PFDD surgery. Clinical improvement was observed in patients that showed improvement of CSF flow after surgery. Based on these results, and according to the literature [36,38,39], a complete brain and spine PC-MRI study, including CSF flow assessment, could be suggested in surgical follow-up, as well as in conservative treatment. Although the selection criteria for PFDD are still mainly based on clinical data and tonsillar ectopia degree, CSF evaluation might better support clinicians in the selection of patients who would be more responsive to surgery. Furthermore, the correlation between CSF flow at the CCJ and post-operative pain/headache relief, in agreement with a prior study [40], highlights that an improvement in CSF flow at the CCJ may precede or coincide with a marked improvement in headache after decompressive surgery.

However, due to the pilot nature of this study, the obtained results should be interpreted with the awareness of some limitations. The first limitation is the retrospective measurement of the fourth ventricle area. To overcome this limitation, as mentioned in the text, all the measurement were performed by a single radiologist; moreover, because this is the first study specifically aimed to describe this new technique, a normal control group was identified and matched to patients by size, sex, and age, as described in details in the Methods section, statistical analysis (“control group selection”). The second limitation of this study is the relatively small sample size of patients. Although our patients were admitted at a single Institution and investigated homogeneously according to a multidisciplinary and standardized protocol, the small cohort could not provide reliable results on the statistical analysis, in primis, on the prognostic role of the presurgical fourth ventricle area. Finally, fourth ventricle area variability was wider in our patients than we expected based on the literature. We are planning to quantify and compare the fourth ventricle volume with the fourth ventricle area in a larger series of patients in a prospective, collaborative, multicentric study in order to better define the predictive role of theses radiological parameters.

In summary, presurgery CSF flow at the CCJ is proposed as a radiological marker that can predict a positive outcome after PFDD in adults with syringomyelia and CM1 at short- and long-term follow-up. Phase-contrast brain MRI should be proposed in the preoperative diagnostic path. Nevertheless, surgical indication and treatment of syringomyelia with CM1 must include careful preoperative analysis of a single clinical case, involving all the specialist figures of the multidisciplinary team.

## Figures and Tables

**Table 1:** CSF flow score and the clinical sign score (CSS).

(a) CSF Flow Score	CSF Flow
0	Regular presence of the flow at the posterior CCJ
1	Slight reduction in CSF flow to the FM
2	CSF flow reduction to the FM
3	CSF flow obstruction to the FM
**(b) CSS Score**	**Motor-Sensory-Sphinter Signs**
0	Absence of motor, sensory AND sphincter signs
1	Presence of motor OR sensory OR sphincter signs
2	Presence of motor AND sensory OR sphincter signs
3	Presence of motor AND sensory AND sphincter signs

(a) The CSF score graduates the CSF flow at the cranio-cervical junction, from 0 (normal) to 3 (absent). (b) The clinical sign score (composed by using motor, sensory, and sphinter signs) is graduated from 0 (no signs) to 3 (all motor, sensory, and sphinter signs). CSF, cerebro-spinal fluid; CCJ, cranio-cervical junction; FM, foramen magnum; CSS, clinical sign score.

**Table 2:** Demographic and clinical variables in the study cohort.

Cohort	All = 36	Percentage (%)	Mean	Range	SD
Sex (f/m)	24/12	66.7/33.3			
Age at surgery (years)			44	18–70	15
Diagnosis (Syr/MC1)	26/10	72.2/27.8			
Surgery (PFDD/PFDDC)	15/21	41.7/58.3			
Tonsillar descent (in mm)			11.2	3–27	6.8
Tonsillar descent cut off (≥5/<5 mm)	30/6	83.3/16.7			
**Symptoms**					
Headache	25	69.4			
Vertigo	19	52.8			
Loss of balance	12	33.33			
Palpitations	12	33.33			
Cervical pain	10	27.8			
Gastrointestinal symptoms	5	13.9			
Excessive sweating	3	8.3			
Syncope	1	2.8			
**Comorbidity**					
Scoliosis	16	44			
Odointoid retroflexion	3	8.3			
Klippel-Feil	2	5.6			
Migraine	2	5.6			
Kabuki Syndrome	1	2.8			
Hearing loss	1	2.8			

Anagraphic, clinical, and comorbidity features of the 36 patients of the cohort. Syr, syringomyelia with CM1; MC1, Chiari malformation type 1; PFDD, posterior fossa decompression with duraplasty; PFDDC, posterior fossa decompression with duraplasty and tonsillar coagulation.

**Table 3:** Radiological and clinical variables: changes at T0-T1, T1-Tlast, T0-Tlast.

T0-T1	Improved	Unchanged	Worsened
Fourth ventricle area	23/35 (66)	0/35 (0)	12/35 (34)
CSF flow score	15/36 (42)	19/36 (53)	2/36 (5)
Vaquero Index	21/26 (80.0)	3/26 (12.0)	2/26 (8.0)
Motor, sensory, sphincter signs (CSS)	9/36 (25.0)	27/36 (75.0)	0/36 (0)
VAS	12/36 (33)	20/36 (56)	4/36 (11)
mRS	5/36 (14)	28/36 (78)	3/36 (8)
**T1-Tlast**			
Fourth ventricle area	21/32 (66)	3/32 (9)	8/32 (25)
CSF flow score	5/32 (16)	24/32 (75)	3/32 (9)
Vaquero Index	16/22 (72)	4/22 (18)	2/22 (9)
CSS	1/32 (3)	29/32 (91)	2/32 (6)
VAS	12/32 (37.5)	12/32 (37.5)	8/32 (25)
mRS	5/32 (16)	24/32 (75)	3/32 (9)
**T0-Tlast**			
Fourth ventricle area	24/32 (75.0)	0/32 (0)	8/32 (25.0)
CSF flow score	16/32 (50.0)	14/32 (43.7)	2/32 (6.3)
Vaquero Index	20/22 (91)	0/22 (0)	2/22 (9)
CSS	9/32 (28)	21/32 (66)	2/32 (6)
VAS	16/32 (50)	8/32 (25)	8/32 (25)
mRS	8/32 (25)	18/32 (56)	6/32 (19)

Clinical and radiological parameters during the follow-up, at T0 (presurgery), at T1 (short-term FU) and at Tlast (long-term FU). VAS, visual analog scale; CSS, clinical sign score; mRS, modified Rankin scale; CSF, cerebro-spinal fluid. Percentages in brackets.

**Table 4:** Fourth ventricle area: patients vs. normal controls at T0 and Tlast.

	Patients				Normal				
	n	Mean	SD	Median	n	Mean	SD	Median	*p*-Value
**Age**	36	43.97	15.07	43.5	36	41.53	17.55	41.5	0.5228
**Sex (m/f)**	12/24				10/26				0.6089
**Fourth Ventricle Area (T0)**									
**All**	36	124.23	52.63	111	36	98.72	24.78	98.5	**0.0438**
**Syr**	26	129.8	57.96	113	36	98.72	24.78	98.5	**0.0328**
**CM1**	10	110.3	34.71	105.5	36	98.72	24.78	98.5	0.3866
**Fourth Ventricle Area (Tlast)**									
**All**	32	110.62	40.27	99.5	36	98.72	24.78	98.5	0.3382
**Syr**	22	116.72	42.92	103.5	36	98.72	24.78	98.5	0.1546
**CM1**	10	97.2	31.5	90.5	36	98.72	24.78	98.5	0.7709

Comparison between patients versus normal controls at T0 (presurgery) and Tlast (post-surgery). At T0, the fourth ventricle area was statistically significant different (All, Syr); at Tlast there were no statistically significant differences between groups. Syr, Syringomyelia with CM1; CM1, Chiari malformation type 1. Bold face characters indicate statistically significant differences.

**Table 5:** Radiological and clinical changes at T0-T1, T0-Tlast, T1-Tlast (statistical comparison).

Parameters	T0	T1	Tlast	*p*-Value		
	Mean (SD)	Mean (SD)	Mean (SD)	T0-T1	T1-Tlast	T0-Tlast
Fourth Ventricle Area (mm^2^)	124.23 (52.63)	116.64 (50.57)	110.63 (40.27)	0.0571	**0.0373**	**0.0093**
CSF flow score	2.3 (0.59)	1.92 (0.65)	1.69 (0.74)	**0.0009**	**0.0325**	**<0.0001**
Vaquero Index	0.49 (0.17)	0.30 (0.18)	0.28 (0.21)	**<0.0001**	0.4196	**0.0002**
CSS	1.19 (0.92)	0.94(0.83)	0.94 (0.88)	**0.0016**	0.572	**0.0091**
VAS	3.29 (2.87)	2.31 (2.58)	1.97 (2.92)	**0.0188**	0.449	**0.0266**
mRS	1.33 (0.53)	1.31 (0.75)	1.28 (0.92)	0.7677	0.4882	0.5

Radiological and clinical variations at pre-surgery and post-surgery follow-up. Mean and SD of all the parameters are reported. All the radiological parameters significantly improved after surgery as well as clinical and pain scores. Bold face characters indicate statistically significant differences. CSF, cerebro-spinal fluid; CSS, clinical sign score; VAS, visual analog scale; mRS: modified Rankin scale.

**Table 6:** Quality of life (SF-36): Comparison between patients and normative Italian sample at T0.

Subscores	Patients	Italian Sample	*p*-Value
Physical Function	65.96 (22.85)	88.69 (14.93)	**<0.001**
Role-Physical	30.77 (36.27)	81.71 (30.27)	**<0.001**
Body Pain	43.46 (24.16)	75.26 (24.07)	**<0.001**
General Health	47.23 (18.17)	66.45 (17.49)	**<0.001**
Vitality	49.42 (21.46)	63.36 (18.19)	**<0.01**
Social Functioning	58.17 (22.34)	78.37 (20.38)	**<0.001**
Role Emotional	47.44 (36.73)	79.20 (33.58)	**<0.001**
Mental Health	61.15 (21.29)	67.76 (18.18)	**0.041**

Statistical comparison (*p*-value) of the SF-36 subscores between the Italian normative sample [32] and the patient group measured at T0 (presurgery). SF-36, the Medical Outcome Study 36-item Short Form Health Survey. Bold face characters indicate statistically significant”.

## Data Availability

Not applicable.
